# Evaluating the Prognostic Factors Associated with Cancer-specific Survival of Differentiated Thyroid Carcinoma Presenting with Distant Metastasis

**DOI:** 10.1245/s10434-012-2711-x

**Published:** 2012-10-28

**Authors:** Brian Hung-Hin Lang, Kai Pun Wong, Chung Yeung Cheung, Koon Yat Wan, Chung-Yau Lo

**Affiliations:** 1 Department of Surgery, The University of Hong Kong, Hong Kong SAR, China; 2 Department of Clinical Oncology, The University of Hong Kong, Hong Kong SAR, China

## Abstract

**Background:**

Because patients with differentiated thyroid carcinoma (DTC) presenting with distant metastasis (DM) have a particularly poor prognosis, examining the prognostic factors in this group is essential. We aimed to evaluate the prognostic factors affecting cancer-specific survival (CSS) in DTC patients presenting with DM.

**Methods:**

Of the 1227 DTC patients, 51 (4.2 %) presented with DM at diagnosis. All patients underwent a total thyroidectomy, followed by radioiodine (RAI) ablation and postablation whole body scan (WBS). Patients were considered to have an osseous metastasis if one of the metastatic sites involved a bone, while RAI avidity was determined by any visual uptake in a known metastatic site on the first WBS. Factors predictive of CSS were determined by univariate and multivariate analyses by the Cox proportional hazard model.

**Results:**

In univariate analysis, older age (relative risk [RR] 1.050, 95 % confidence interval [CI] 1.010–1.091, *P* = 0.014), DM discovered before WBS (RR 3.401, 95 % CI 1.127–10.309, *P* = 0.030), follicular thyroid carcinoma (RR 3.095, 95 % CI 1.168–8.205, *P* = 0.025), osseous metastasis (RR 4.695, 95 % CI 1.379–15.873, *P* = 0.013), non-RAI avidity (RR 3.355, 95 % CI 1.280–8.772, *P* = 0.014), and external beam radiotherapy to DM (RR 3.241, 95 % CI 1.093–9.614, *P* = 0.034) were significant poor prognostic factors for CSS. In the multivariate analysis, after adjusting for other factors, osseous metastasis (RR 6.849, 95 % CI 1.495–31.250, *P* = 0.013) and non-RAI avidity (RR 7.752, 95 % CI 2.198–27.027, *P* = 0.001) were the two independent poor prognostic factors for CSS. Older age almost reached statistically significance (RR 1.055, 95 % CI 0.996–1.117, *P* = 0.068).

**Conclusions:**

DTC patients presenting with DM accounted for 4.2 % of all patients. Because osseous metastasis and RAI avidity were independent prognostic factors, future therapy should be directed at improving the treatment efficacy of osseous and/or non-RAI-avid metastases.

Differentiated thyroid carcinoma (DTC) accounts for over 90 % of all follicular cell–derived thyroid malignancies and comprises two distinct histological types, papillary thyroid carcinoma and follicular thyroid carcinoma.^1^ Despite the rising incidence, its prognosis remains excellent, with a 10-year cancer-specific survival (CSS) of up to 90 %.^2^,^3^ However, up to 9 % of DTC patients present with distant metastasis (DM) at the time of diagnosis.^4^ Unlike the rest of the group, this specific group carries a far worse prognosis.^2^ In fact, this group accounts for much of the overall cancer-specific deaths.^2^,^3^

Although previous studies have identified a number of prognostic factors significantly associated with poorer CSS, such as age >45 years, poor differentiation, follicular thyroid carcinoma, Hürthle cell variant, iodine avidity, incomplete local control, and extrapulmonary DM, some of these studies tended to group and analyze those with DM at diagnosis and those with DM discovered some time after initial treatment together.^4^–^14^ It is recognized both groups differ in their prognosis and treatment response, and so they ideally should be considered separately.^4^,^8^,^11^ Furthermore, both the prognostic factors and the outcomes varied between studies.^4^,^8^,^9^,^11^ Apart from possible referral differences, some studies included patients who were initially managed outside their primary institution. This may pose a selection bias because those with more advanced or extensive disease and/or with poorer response to initial treatment were more likely referred to a tertiary referral institution.

To minimize patient heterogeneity, the present study aimed to select only patients who had surgery, subsequent radioiodine (RAI), and whole body scan (WBS) at our institution. We aimed to review our single-institution experience and to specifically evaluate the prognostic factors associated with CSS of DTC patients with DM at diagnosis.

## Patients and Methods

### Patients

The present study protocol was approved by the local institutional review board. From 1986 to 2010, a total of 1227 patients underwent surgery for histologically proven DTC. Of these, 51 patients (4.2 %) were found to have DM at diagnosis. These were the patients whose DM was identified any time before surgery or at the time of the first postablation WBS (i.e., 2–3 months after surgery). Patients who underwent primary thyroid surgery, RAI, or WBS performed outside our institution or those who had their DM discovered after the first WBS were excluded. Also, if the primary tumor contained a poorly differentiated or anaplastic component, it was excluded.

### Treatment Protocol

Details of the treatment and follow-up protocols have been described previously.^2^ Total thyroidectomy was preferred. Simultaneous central (level VI) with or without lateral (levels II–V) selective neck dissection was performed for clinically proven cervical lymph node metastasis. Surgical metastasectomy was considered if the metastasis was solitary and resectable. Two months after initial surgery, a standard ablative RAI dose of 3 GBq or 80 mCi was provided after levothyroxine (LT4) withdrawal or with recombinant thyroid-stimulating hormone (rTSH). TSH-suppressive LT4 treatment was commenced immediately afterward. This was followed by the postablation WBS or posttherapy scan 4–7 days later. The subsequent therapy for DM usually comprised 5.5 GBq (or 150 mCi) RAI and was administered periodically every 6 months until no more uptake was observed on WBS or until disease progressed despite treatment. External beam radiotherapy (EBRT) to the neck was provided to patients with extensive extrathyroidal tumor extension, incomplete resection, and/or extracapsular lymph node metastasis (40 Gy over 3 weeks for adjuvant treatment and 50 Gy over 4 weeks for gross residual disease). In some cases of incomplete resection or gross residual tumor after surgery, EBRT might be provided before RAI because it is more effective in controlling gross residual disease.^15^ EBRT to metastatic sites was performed for symptomatic and unresectable bone or brain lesions, such as vertebral metastases resulting in neurological or compressive symptoms that were not amenable to surgery. All treatment decisions were made after discussion at a multidisciplinary tumor board meeting. Although the above protocol was followed, individual patients’ preference and performance status were considered.

## Methods

All data, including disease status and cause of death, were prospectively collected after 1995. The diagnosis of DM was based on clinical and/or positive findings on WBS, radiography (e.g., chest x-ray), CT scan, MRI, or tissue biopsy. Iodine avidity was determined by visual uptake in the known site of metastatic disease by WBS after the first RAI ablation under hypothyroid conditions. Absence of visual uptake on WBS was defined as disease being non-RAI avid. Sites of DM were classified into pulmonary versus extrapulmonary and into osseous versus nonosseous. For multiple DM sites, if one of the sites involved the lung or bone, the patient would still be classified into pulmonary or osseous, respectively. The latest disease status was classified into progressive disease, static disease, or remission on the basis of the serum thyroglobulin (Tg) trend and/or the changes in the size and number of metastases by diagnostic WBS or axial imaging. Patients were classified as having died of DTC if this was stated as the primary cause of death or as a contributing cause of death in the death certificate. In patients in whom the cause of death was uncertain, the patient was assumed to have died from DTC if extensive progressive metastatic disease was present at the last follow-up.

### Tg Measurement

Stimulated Tg (sTg) was defined as a Tg level measured in the presence of TSH >30 mIU/L either by 4-week LT4 withdrawal or rTSH injections. The preablation sTg level was assessed just before the RAI ablation, and the postablation sTg was assessed 6 months after RAI ablation. Tg levels were measured in the same laboratory, although less sensitive biochemical assays were used before the year 2006.^16^ After that, the assay used was the Immulite 2000 (Diagnostic Products, Roche, Los Angeles, CA). This was calibrated against the CRM-457 standard. Normal reference range was <0.5–55 μg/L, and sensitivity was <0.2 μg/L.

### Statistical Analysis

Statistical analysis was performed by Chi-square or Fisher’s exact test to compare categorical variables, and the Mann–Whitney *U*-test was used to compare continuous variables between groups. Continuous variables were expressed as medians with ranges. Survival was calculated from the diagnosis of DM to the date of last follow-up or death. CSS was calculated by the Kaplan–Meier method, and survival outcomes were compared by the log rank test. Factors predictive of CSS were determined by univariate and multivariate analyses by the Cox proportional hazard model. All statistical analyses were performed by SPSS software, version 18.0 (SPSS, Chicago, IL).

## Results

Table 1 shows the baseline characteristics of the cohort. DM was identified before the first postablation WBS in 38 patients (74.5 %) and by first postablation WBS in 13 patients (25.5 %). Of the 38 patients diagnosed before WBS, 20 were asymptomatic, while the other 18 had symptoms/signs as a result of the DM. These symptoms/signs included bilateral lower limb weakness (*n* = 3), neck skin nodules (*n* = 2), respiratory complaints (*n* = 3), rib pain (*n* = 1), back pain (*n* = 1), generalized bone pain (*n* = 2), long bone fracture (*n* = 1), pelvic mass (*n* = 1), scalp mass (*n* = 2), seizure (*n* = 1), and sternal swelling (*n* = 1). Of the 20 asymptomatic patients, 15 patients had visible lung masses on plain preoperative chest x-ray, while the other 5 patients had bone and/or lung metastases detected on preoperative CT scan.

**Table 1:** Baseline characteristics of the 51 patients with differentiated thyroid carcinoma presenting with distant metastasis

Characteristic	Value^a^
Age at diagnosis of distant metastasis (y)	62.1 (12.2–75.1)
Gender	
Male	15 (29.4)
Female	36 (70.6)
Discovery of distant metastasis	
At diagnosis of primary tumor	38 (74.5)
Asymptomatic	20 (39.2)
Symptomatic	18 (35.3)
At first postablation whole body scan	13 (25.5)
Type and extent of initial operation	
Total thyroidectomy	23 (45.1)
Total thyroidectomy + CND	2 (3.9)
Total thyroidectomy + CND + metastasectomy	3 (5.9)
Total thyroidectomy + SND	22 (43.1)
Total thyroidectomy + SND + metastasectomy	1 (2.0)
Histopathology	
Papillary thyroid carcinoma	34 (66.7)
Follicular thyroid carcinoma	17 (33.3)
Primary tumor characteristics	
Size (cm)	3.0 (0.5–12.0)
Extrathyroidal extension	26 (51.0)
Tumor multifocality	25 (49.0)
Cervical lymph node metastases (pN1)	32 (62.7)
Postoperative treatment	
Radioiodine ablation/therapy	51 (100)
External radiotherapy to neck region	14 (27.5)
Provided before radioiodine	4 (7.8)
Provided after radioiodine	10 (19.6)
External radiotherapy to distant metastasis	22 (43.1)
Preablation stimulated thyroglobulin (ng/mL)	1478 (2.7 to > 4800)
Postablation stimulated thyroglobulin (ng/mL)	788.5 (0.2 to > 4800)
Need for reoperative neck dissection	7 (13.7)
Time interval from initial operation to neck reoperation (mo)	59.3 (12.4–63.9)

*CND* central neck dissection, *SND* selective neck dissection

^a^Data are presented as median (range) or *n* (%)

Ten patients (19.6 %) underwent surgical metastasectomy. Three patients had metastases removed before initial thyroid surgery: one underwent a craniotomy for a solitary brain metastasis, while the other two underwent excision of spinal bone metastasis, spinal cord decompression, and fixation. Four patients had concomitant metastasectomy at the time of the thyroid surgery: two underwent excision of neck skin metastases, and two underwent en bloc manubrial and clavicular excision for bone and mediastinal metastases. Three patients required metastasectomy after the thyroid surgery: one presented with spinal cord compression from a spinal metastasis 10 days after thyroid surgery and so required excision of the vertebral metastasis, spinal cord decompression, and fixation. The other two patients underwent excision of skeletal solitary metastasis involving the paraspinal muscle. Twenty-eight patients (54.9 %) had single-site DM (lung in 18 patients, bone in 7 patients, brain in 3 patients). The other 23 patients had more than one metastatic site, including lung and bone (*n* = 12), lung and mediastinum (*n* = 3), lung and brain (*n* = 4), lung and skin (*n* = 2), and lung and scalp (*n* = 2).

After surgery, all patients received one ablative dose of 3–5.5 GBq RAI. The median cumulative RAI dose was 11.0 (3.0–27.5) GBq. The median cumulative dose was significantly higher in RAI-avid DM than non-RAI-avid DM (11.0 vs. 5.5 GBq, *P* = 0.006). In our cohort, despite good tumor response (as judged by a combination of decreasing Tg and radiological response), 4 patients refused further RAI therapy after ablation. Two patients failed to complete EBRT to DM (one for brain metastasis and one for bone metastasis). Of the 14 patients who received EBRT, 4 received EBRT before RAI ablation because they were considered to have gross residual disease left in their thyroid bed and so to be at increased risk of locoregional recurrence. The median follow-up was 100.4 (range 6.6–229.1) months. At the time of analysis, 18 (35.3 %) died of DTC and 2 (3.9 %) died of an unrelated cause. Of the 31 patients still alive, 14 had progressive disease, 12 had static disease, and 5 were in remission. The 5-year and 10-year overall survival were 85.4 % and 52.4 %, respectively, while the 5-year and 10-year CSS were 87.6 % and 58.7 %, respectively.

Table 2 shows the univariate analysis of CSS by the Cox proportional hazard model. Older age at diagnosis (relative risk [RR] 1.050, 95 % confidence interval [CI] 1.010–1.091, *P* = 0.014), DM discovered at presentation (RR 3.401, 95 % CI 1.127–10.309, *P* = 0.030), follicular thyroid carcinoma (RR 3.095, 95 % CI 1.168–8.205, *P* = 0.025), osseous metastasis (RR 4.695, 95 % CI 1.379–15.873, *P* = 0.013), non-RAI avidity (RR 3.355, 95 % CI 1.280–8.772, *P* = 0.014), and EBRT to DM (RR 3.241, 95 % CI 1.093–9.614, *P* = 0.034) were statistically significant poor prognostic factors for CSS.

**Table 2:** Univariate Cox regression analysis of in differentiated thyroid carcinoma patients presenting with distant metastasis

Variable	*n* (%)	Relative risk (95 % CI)	*P*
Age at diagnosis of distant metastasis (y)	51 (100)	1.050 (1.010–1.091)	0.014*
Sex			0.438
Male	15 (29.4)	1	
Female	36 (70.6)	1.543 (0.515–4.630)	
Discovery of distant metastasis			0.030*
At diagnosis or before surgery	38 (74.5)	1	
At postablation whole body scan	13 (25.5)	0.294 (0.097–0.887)	
Primary tumor size (cm)	51 (100)	1.126 (0.840–1.509)	0.470
Histopathology			0.023*
Papillary thyroid carcinoma	34 (66.7)	1	
Follicular thyroid carcinoma	17 (33.3)	3.095 (1.168–8.205)	
Extrathyroidal extension			0.150
No	25 (49.0)	1	
Yes	26 (51.0)	2.210 (0.751–6.504)	
Tumor multifocality			0.360
No	26 (51.0)	1	
Yes	25 (49.0)	1.572 (0.597–4.142)	
Tumor bilaterality			0.799
No	38 (74.5)	1	
Yes	13 (25.5)	1.159 (0.371–3.619)	
Cervical lymph node metastasis			0.166
No	19 (37.3)	1	
Yes	32 (62.7)	1.972 (0.754–5.155)	
Completeness of primary operation			0.956
Complete	39 (76.5)	1	
Incomplete	12 (23.5)	1.031 (0.347–3.068)	
Metastasectomy			0.967
No	41 (80.4)	1	
Yes	10 (19.6)	1.064 (0.228–4.673)	
Need for neck reoperation			0.483
No	44 (86.3)	1	
Yes	7 (13.7)	1.704 (0.384–17.576)	
Stimulated thyroglobulin (ng/mL)			
Before ablation	51 (100.0)	1.000 (0.999–1.001)	0.373
After ablation	51 (100.0)	1.000 (0.999–1.001)	0.580
Site of distant metastasis			0.256
Pulmonary	41 (80.4)	1	
Extrapulmonary	10 (19.6)	3.241 (0.880–7.631)	
Site of distant metastasis			0.008*
Nonosseous	30 (58.8)	1	
Osseous	21 (41.2)	3.788 (1.414–10.101)	
No. of DM sites			0.085
Single	28 (54.9)	1	
Two or more	23 (45.1)	2.376 (0.888–6.358)	
Radioiodine avidity^a^			0.014*
Yes	37 (72.5)	1	
No	14 (27.5)	3.355 (1.280–8.772)	
Cumulative radioiodine activity (GBq)	51 (100)	0.944 (0.859–1.037)	0.230
EBRT to the neck region			0.665
No	37 (72.5)	1	
Yes	14 (27.5)	1.242 (0.465–3.316)	
EBRT to distant metastasis			0.034*
No	29 (56.9)	1	
Yes	22 (43.1)	3.241 (1.093–9.614)	

*CI* confidence interval, *DM* distant metastasis, *EBRT* external beam radiotherapy

^a^Based on the finding of the first postablation whole body scan

* Statistically significant (*P* < 0.05)

Table 3 shows the multivariate analysis of CSS by the Cox proportional hazard model. After adjusting for other significant factors such as age at diagnosis (*P* = 0.068), timing of DM (*P* = 0.792), histopathology (*P* = 0.766), and EBRT to DM (*P* = 0.413), osseous metastasis (RR 6.849, 95 % CI 1.495–31.250, *P* = 0.013) and non-RAI avidity (RR 7.752, 95 % CI 2.198–27.027, *P* = 0.001) were independent poor prognostic factors for CSS. Age at diagnosis almost reached statistical significance (RR 1.055, 95 % CI 0.996–1.117, *P* = 0.068).

**Table 3:** Multivariate analysis for CSS in differentiated thyroid carcinoma patients presenting with distant metastasis

Covariate	β coefficient	Relative risk (95 % CI)	*p*
Age at diagnosis of distant metastasis (y)	0.053	1.055 (0.996–1.117)	0.068
Discovery of distant metastasis			0.792
At diagnosis or before surgery		1	
At postablation whole body scan	0.234	0.791 (0.139–4.494)	
Histopathology			0.766
Papillary thyroid carcinoma		1	
Follicular thyroid carcinoma	0.226	1.255 (0.283–5.559)	
Site of distant metastasis			0.013*
Nonosseous		1	
Osseous	1.922	6.849 (1.495–31.250)	
Radioiodine avidity^a^			0.001*
Yes		1	
No	2.044	7.752 (2.198–27.027)	
EBRT to distant metastasis			0.413
No		1	
Yes	0.530	1.699 (0.477–6.050)	

*CSS* cancer-specific survival, *CI* confidence interval, *ERBT* external beam radiotherapy

^a^Based on the finding of the postablation whole body scan

* Statistically significant (*P* < 0.05)

**Fig. 1:**
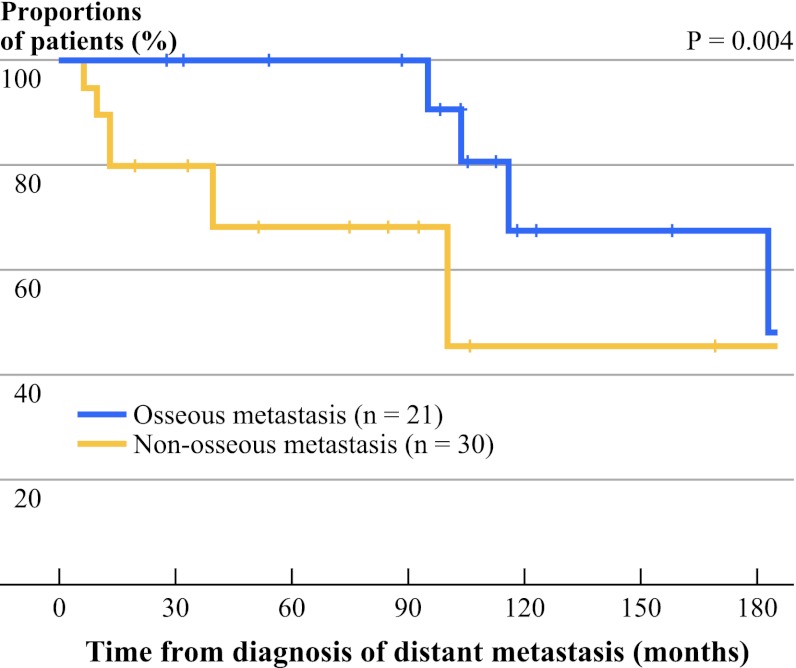
Cancer-specific survival curves between those with osseous metastasis (*n* = 21) and those with nonosseous metastasis (*n* = 30)

Figure 1 shows the CSS survival curves between those with osseous metastasis and with nonosseous metastasis. The 5-year and 10-year CSS between patients with osseous metastasis and with nonosseous metastasis were 68.6 % and 45.7 % vs. 100 % and 67.6 %, respectively (*P* = 0.004).

**Fig. 2:**
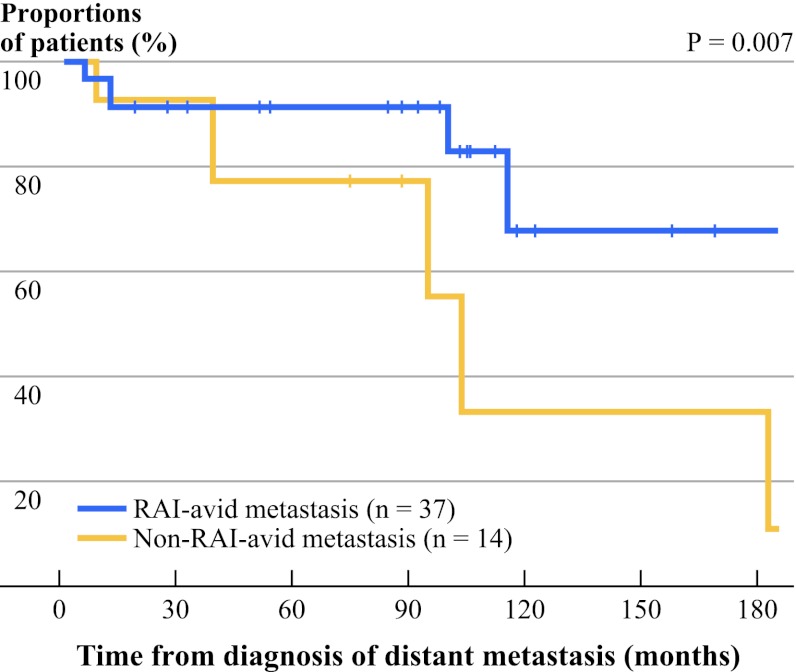
Cancer-specific survival curves between those with radioiodine (RAI)-avid distant metastasis (*n* = 37) and those with non-RAI-avid distant metastasis (*n* = 14)

Figure 2 shows the CSS survival curves between those with RAI-avid DM and with non-RAI-avid DM. The 5-year and 10-year CSS between patients with RAI-avid DM and with non-RAI-avid DM were 91.7 % and 67.9 % vs. 77.4 % and 33.2 %, respectively (*P* = 0.007).

## Discussion

This study represented our 25-year single-institution experience of managing DTC presenting with DM and was aimed at examining prognostic factors associated with this cohort. Unlike previous reports, we excluded those who developed DM after the first WBS.^4^–^14^ This was important because first, the prognosis of those discovered before or by WBS is different from those discovered after WBS.^4^,^12^ Second, some DM are believed to be a result of locoregional disease progression (such as disease progressed from the central neck to the mediastinum) and so might represent a different disease altogether.^4^,^8^,^9^,^11^ Third, those discovered before or by WBS are often considered more “RAI naive” and so they are generally more RAI responsive and have higher RAI avidity.^4^,^8^

Similar to previous studies, our data confirmed that osseous metastasis and non-RAI-avid DM were independent poor prognostic factors for CSS.^6^,^7^,^9^ In one of the largest series, Durante et al. reported that those with osseous metastasis had significantly poorer 10-year overall survival than those with pulmonary metastasis (25 % vs. 63 %, *P* < 0.001).^6^ Similar observations had been made in other studies.^11^–^14^,^17^,^18^ Ideally, our analysis could have been improved by selecting a group with osseous metastasis only, but because the number of these patients was so small (*n* = 7), it was difficult to do so. One explanation why osseous metastasis had a poorer survival outcome might be because RAI is generally less effective for osseous metastasis.^5^,^17^,^18^ In our opinion, adopting an aggressive surgical approach by resecting solitary osseous metastasis may improve the prognosis of this group. However, because not all osseous metastases are amenable to surgical resection, other systematic treatment options, such as intravenous bisphosphonates and new targeted drugs, may be important and should be considered together with RAI and EBRT.^17^,^18^

RAI avidity was found to be the other independent prognostic factor for CSS, a finding consistent with other studies.^4^,^9^,^10^ The reasons why RAI avidity might have been a significant prognostic factor are 2-fold. First, non-RAI-avid DM is generally less differentiated and so behaves more aggressively.^19^–^21^ Second, non-RAI-avid DM are less responsive to RAI therapy.^21^ RAI remains the most effective treatment for DM and could achieve long-lasting remission in young patients.^5^,^6^ In our study, the 5 patients in remission were relatively young. They were 12.2 to 59.3 years old at diagnosis. Although toward the latter period some patients with negative WBS did undergo F-18-fluorodeoxyglucose–positron emission tomography (FDG-PET) to further delineate their DM, the FDG-PET data were not available at the time of analysis, and so the prognostic value of FDG positivity could not be assessed. Nevertheless, because RAI avidity was a significant factor, findings on the FDG-PET scan probably carry significant prognostic value, as there is often a flip-flop phenomenon between RAI and FDG uptakes.^20^,^22^

Although previous studies found age at DM to be a significant factor, it did not reach statistical significant (*P* = 0.068) in our multivariate analysis.^8^–^12^,^14^ Perhaps with a larger sample size it might become significant. From our data, 4 patients (30.8 %) aged <45 years eventually died of DTC, while 14 patients (36.8 %) aged ≥45 years died of DTC. Other previously identified significant factors like incomplete local control or postsurgical sTg level were not significant in this study.^13^,^14^ Perhaps these findings imply that the treatment priority for DM should be on distant disease control rather than on local disease control. Although these findings appeared to be in discordant with some studies that found a significant correlation between adequate local control and improved survival outcome, they were consistent with the recommended treatment guidelines for metastatic DTC.^1^,^14^ One of the main shortcomings of our study was that the number of patients was relatively small, and so some insignificant findings might have been related to the inadequate power of the study. Because we defined RAI avidity as visual uptake in the known site of metastatic disease by WBS after the first RAI ablation, we did not fully account for some patients who might have an initial positive postablation WBS and a subsequent negative diagnostic WBS, or some patients with mixed RAI-avid and non-RAI-avid DM.

DTC patients presenting with DM at diagnosis accounted for 4.5 % of all patients treated at our institution. Patients with osseous metastasis had a significantly worse CSS than those with nonosseous metastasis, while patients with non-RAI-avid metastasis also had a significantly worse CSS than those with RAI-avid metastasis. Both osseous metastasis and RAI avidity were independent prognostic factors for CSS in DTC patients presenting with DM. To further improve the overall prognosis, future therapy should be directed at improving the efficacy of treatment of patients with osseous and/or non-RAI-avid metastases.

## References

[1] Cooper DS, Doherty GM, Hauger BR (2009). Revised American Thyroid Association management guidelines for patients with thyroid nodules and differentiated thyroid cancer. Thyroid..

[2] Lang BH, Lo CY, Chan WF, Lam KY, Wan KY (2007). Staging systems for papillary thyroid carcinoma: a review and comparison. Ann Surg..

[3] Lang BH, Lo CY, Chan WF, Lam KY, Wan KY (2007). Prognostic factors in papillary and follicular thyroid carcinoma: implications for cancer staging. Ann Surg Oncol..

[4] Lee J, Soh EY (2010). Differentiated thyroid carcinoma presenting with distant metastasis at initial diagnosis clinical outcomes and prognostic factors. Ann Surg..

[5] Dinneen SF, Valimaki MJ, Bergstralh EJ, Goellner JR, Gorman CA, Hay ID (1995). Distant metastases in papillary thyroid carcinoma:100 cases observed at one institution during 5 decades. J Clin Endocrinol Metab..

[6] Durante C, Haddy N, Baudin E (2006). Long-term outcome of 444 patients with distant metastases from papillary and follicular thyroid carcinoma: benefits and limits of radioiodine therapy. J Clin Endocrinol Metab..

[7] Lin JD, Huang MJ, Juang JH (1999). Factors related to the survival of papillary and follicular thyroid carcinoma patients with distant metastases. Thyroid..

[8] Haq M, Harmer C (2005). Differentiated thyroid carcinoma with distant metastases at presentation: prognostic factors and outcome. Clin Endocrinol (Oxf)..

[9] Sampson E, Brierley JD, Le LW, Rotstein L, Tsang RW (2007). Clinical management and outcome of papillary and follicular (differentiated) thyroid cancer presenting with distant metastasis at diagnosis. Cancer..

[10] Mihailovic J, Stefanovic L, Malesevic M, Markoski B (2009). The importance of age over radioiodine avidity as a prognostic factor in differentiated thyroid carcinoma with distant metastases. Thyroid..

[11] Nixon IJ, Whitcher MM, Palmer FL (2012). The impact of distant metastases at presentation on prognosis in patients with differentiated carcinoma of the thyroid gland. Thyroid..

[12] Shoup M, Stojadinovic A, Nissan A (2003). Prognostic indicators of outcomes in patients with distant metastases from differentiated thyroid carcinoma. J Am Coll Surg..

[13] Huang IC, Chou FF, Liu RT (2012). Long-term outcomes of distant metastasis from differentiated thyroid carcinoma. Clin Endocrinol (Oxf)..

[14] Sugitani I, Fujimoto Y, Yamamoto N (2008). Papillary thyroid carcinoma with distant metastases: survival predictors and the importance of local control. Surgery..

[15] Chow SM, Yau S, Kwan CK, Poon PC, Law SC (2006). Local and regional control in patients with papillary thyroid carcinoma: specific indications of external radiotherapy and radioactive iodine according to T and N categories in AJCC 6th edition. Endocr Relat Cancer..

[16] Wong H, Wong KP, Yau T (2012). Is there a role for unstimulated thyroglobulin velocity in predicting recurrence in papillary thyroid carcinoma patients with detectable thyroglobulin after radioiodine ablation?. Ann Surg Oncol..

[17] Orita Y, Sugitani I, Matsuura M (2010). Prognostic factors and the therapeutic strategy for patients with bone metastasis from differentiated thyroid carcinoma. Surgery..

[18] Haugen BR, Kane MA (2010). Approach to the thyroid cancer patient with extracervical metastases. J Clin Endocrinol Metab..

[19] Lang BH, Law TT (2011). The role of 18F-fluorodeoxyglucose positron emission tomography in thyroid neoplasms. Oncologist..

[20] Robbins RJ, Wan Q, Grewal RK (2006). Realtime prognosis for metastatic thyroid carcinoma based on 2-[^18^F]fluoro-2-deoxy-d-glucose-positron emission tomography scanning. J Clin Endocrinol Metab..

[21] Kim WG, Ryu JS, Kim EY (2010). Empiric high-dose 131-iodine therapy lacks efficacy for treated papillary thyroid cancer patients with detectable serum thyroglobulin, but negative cervical sonography and 18F-fluorodeoxyglucose positron emission tomography scan. J Clin Endocrinol Metab..

[22] Vitale G, Fonderico F, Martignetti A (2001). Pamidronate improves the quality of life and induces clinical remission of bone metastases in patients with thyroid cancer. Br J Cancer..

